# Development and validation of a nomogram to predict atelectasis in adult lymph node fistula tracheobronchial tuberculosis patients

**DOI:** 10.3389/fmed.2025.1637007

**Published:** 2025-08-07

**Authors:** Quhua Yin, Guojian Ou, Yi Zhou, Xiaojian Wen, Heping Huang, Jie Ling, Li Luo

**Affiliations:** ^1^Department of Radiology, Hunan Chest Hospital, Changsha, China; ^2^Endoscopy Center, Hunan Chest Hospital, Changsha, China

**Keywords:** tuberculosis, bronchial diseases, lymph node fistula, atelectasis, nomogram, prediction model

## Abstract

**Background:**

Lymph node fistula tracheobronchial tuberculosis (TBTB) is a severe respiratory condition that can result in complications such as airway stenosis and atelectasis, posing significant clinical challenges, particularly in adults. Currently, no standardized assessment tools are available to predict the risk of atelectasis in these patients, highlighting the need to develop an effective predictive model to guide early clinical intervention and personalized treatment.

**Methods:**

A retrospective study was conducted involving 547 adult patients diagnosed with lymph node fistula TBTB at our hospital between January 2017 and December 2023. Diagnoses were confirmed by chest computed tomography, bronchoscopy, and combined etiological or pathological examinations. After applying the inclusion and exclusion criteria, 301 cases were included in the final analysis. Patients were randomly assigned to a development group (*n* = 211, 70%) and a validation group. Following univariate and multivariable logistic regression to identify significant predictors, we developed a nomogram. Model validation included assessment of discriminatory ability [receiver operator characteristic (ROC) analysis], calibration accuracy, and clinical utility (DCA).

**Results:**

Among the 301 patients with lymph node fistula TBTB, the incidence of atelectasis was 60.13% (181/301). Of those, 72.93% (132/181) had right lung involvement, and 50.28% (91/181) specifically had atelectasis in the right middle lobe. Independent predictors identified by multivariable logistic regression included age, occupation as a farmer, mediastinal lymphadenopathy with ring enhancement, and right middle lobe bronchial involvement. A risk nomogram was developed using these predictors. The area under the curve (AUC) of the nomogram was 0.824 (95% CI: 0.685–0.806) in the development group and 0.857 (95% CI: 0.702–0.877) in the validation group. Calibration plots based on 500 bootstrap resamples showed good agreement between predicted and observed probabilities across both groups. DCA revealed that the model provided a net clinical benefit within threshold probability ranges of 0.2–0.9 for the development group and 0.15–0.85 for the validation group.

**Conclusion:**

The predictive model and associated nomogram developed in this study can accurately estimate the risk of atelectasis in adult patients with lymph node fistula TBTB. This tool may assist clinicians in developing individualized intervention strategies.

## 1 Introduction

Tracheobronchial tuberculosis (TBTB) is a chronic inflammatory condition caused by *Mycobacterium tuberculosis* infection of the airway mucosa and submucosal layers (1). Progressive mural thickening, luminal narrowing, and eventual airway occlusion can lead to atelectasis. Once atelectasis occurs, a cycle of infection, obstruction, and reinfection may develop (2, 3), resulting in impaired ventilation and gas exchange, reduced exercise tolerance, and, in severe cases, respiratory failure and multiple organ dysfunction (4, 5). Among TBTB subtypes, lymph node fistula TBTB warrants particular attention due to the rupture of mediastinal or hilar lymph nodes into the airways, resulting in fistula formation (3). This subtype is often associated with serious complications, including bronchial obstruction and atelectasis (6). Compared with pediatric cases, adult cases are generally more complex, frequently involving additional complications and requiring individualized management. Furthermore, adults tend to have more extensive social interactions, contributing to longer transmission chains and substantial public health implications (7).

The presence of atelectasis in TBTB introduces several clinical challenges. First, although previous studies have identified associations between older age, delayed or incorrect diagnosis, and the absence of timely bronchoscopic intervention with atelectasis development (7), no risk stratification tool is currently available. This limitation prevents clinicians from identifying the optimal timing for intervention. Second, fibrotic airway narrowing progresses rapidly, leaving a narrow therapeutic window. Patients often require repeated balloon dilations or stent placements, yet restenosis rates remain high (2). Third, even after successful airway reopening, re-expansion of the affected lung segment is often slow, and persistent ventilation–perfusion mismatches significantly impair quality of life (3, 8). Finally, prolonged bacillary shedding and frequent medical visits in adult patients exacerbate tuberculosis transmission and increase the economic burden (9). These factors highlight the urgent need for an early detection framework and precision management strategy.

In this context, we aimed to develop and internally validate a predictive model for atelectasis in adult patients with lymph node fistula TBTB by integrating multiple variables, including age, occupation, clinical symptoms, computed tomography (CT) findings, and sputum test results. We further constructed an easy-to-use nomogram to enable early identification of high-risk individuals. The goal was to support timely bronchoscopic intervention and individualized anti-tuberculosis therapy, thereby reducing atelectasis incidence, preserving lung function and prognosis, and mitigating tuberculosis transmission and its societal burden.

## 2 Materials and methods

### 2.1 Patients

**FIGURE 1:**
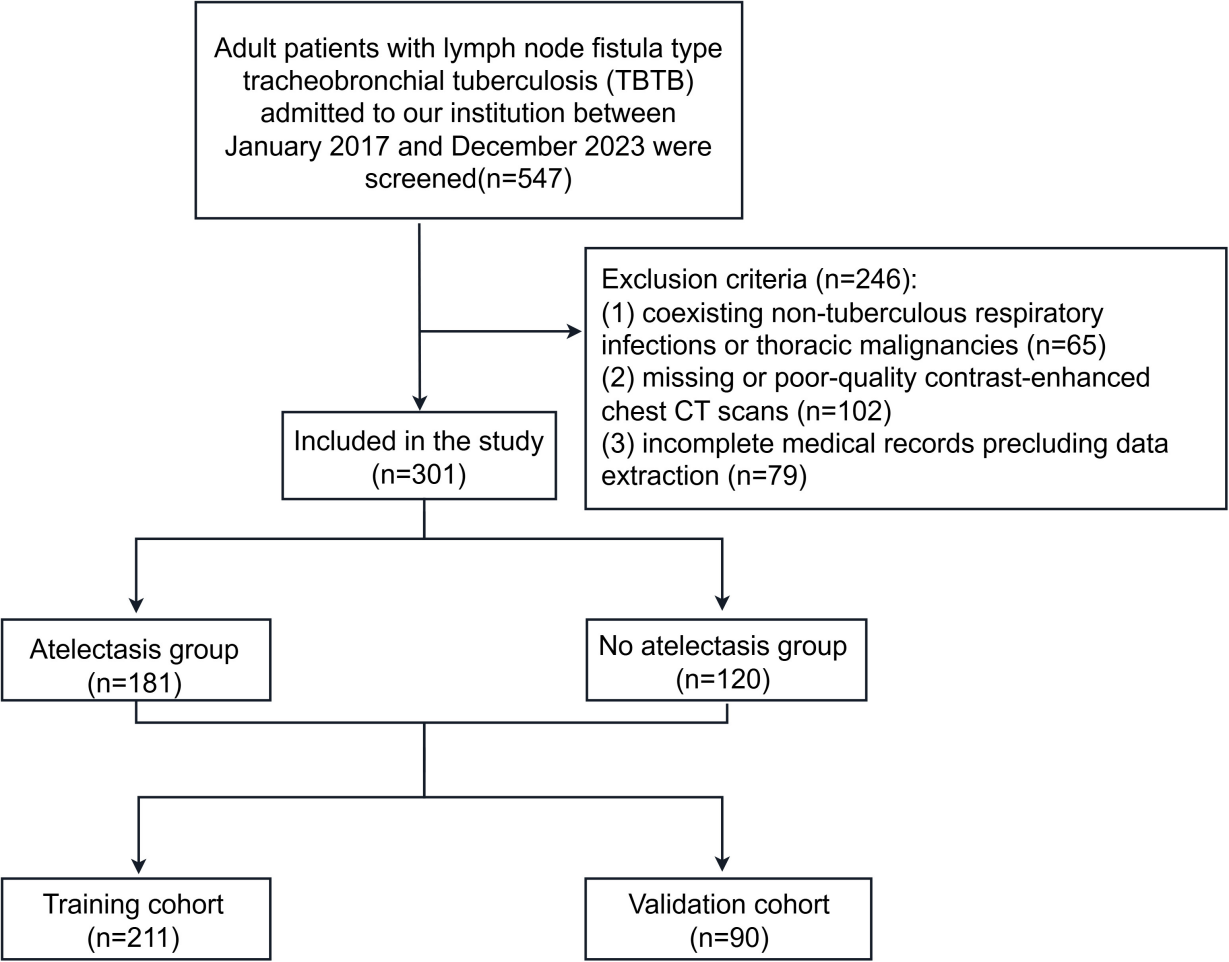
Flow diagram showing patient enrollment.

A retrospective study was conducted including 547 adult patients diagnosed with lymph node fistula TBTB between January 2017 and December 2023. Diagnoses were confirmed by chest CT, bronchoscopy, and combined microbiological or pathological examinations (Figure 1). Inclusion criteria were: (1) adulthood (≥18 years); and (2) a verified diagnosis of bronchial tuberculosis with lymphatic fistula subtype determination based on the WS288-2017 Diagnostic Criteria for Pulmonary Tuberculosis (10) and the Guidelines for the Diagnosis and Treatment of Tracheobronchial Tuberculosis (Trial) (11); and (3) availability of complete demographic, clinical, radiological, and laboratory records. Exclusion criteria included: (1) coexisting non-tuberculous respiratory infections or thoracic malignancies; (2) missing or poor-quality contrast-enhanced chest CT scans; and (3) incomplete medical records precluding data extraction.

### 2.2 Data collection and radiological assessment

Demographic information (age, sex, and occupation), presenting symptoms (cough, sputum production, fever, and hemoptysis), and sputum test results (smear and culture) were extracted from the hospital’s electronic medical record system. Contrast-enhanced chest CT scans were independently reviewed by two experienced thoracic radiologists, blinded to clinical data. The reviewers recorded the presence of cavity formation, the lobe or segment affected by atelectasis, bronchial involvement site, enhancement pattern of enlarged mediastinal or hilar lymph nodes, lymph node calcification, and the visibility of any fistulous opening. Discrepancies between reviewers were resolved through consensus discussion with a senior thoracic radiologist. Atelectasis was defined radiologically as partial or complete collapse of a lung lobe or segment with associated loss of normal aeration and expansion.

### 2.3 Statistical analysis

Continuous variables that satisfied normality are reported as mean ± SD and contrasted through independent *t*-testing. Variables lacking normal distribution are given as median with interquartile range (Q_1_–Q_3_) and analyzed using the Kruskal–Wallis test. Categorical outcomes are summarized as frequency and proportion, with between-group differences evaluated by the Chi-square test or, when expected frequencies were <10, Fisher’s exact test.

Participants were randomly split in a 7: 3 ratio into development and validation cohorts (Figure 1). Univariate logistic regression isolated variables associated with atelectasis (*P* < 0.05). These candidates were subsequently entered into a multivariable logistic model to determine independent determinants, all of which were retained for construction of a predictive nomogram.

Model discrimination was quantified via the area under the receiver operator characteristic (ROC) curve. Internal validity and predictive consistency were tested through 500 bootstrap replications. Calibration performance was illustrated by overlaying predicted probabilities on actual event rates, and clinical applicability was judged with decision-curve analysis across a spectrum of thresholds. All statistical procedures employed EmpowerStats and R version 4.1.2, with two-sided *P* < 0.05 indicating significance.

## 3 Results

### 3.1 Patient characteristics

This study included 301 adult patients with lymph node fistula TBTB, consisting of 130 males and 171 females, with ages ranging from 18 to 85 years and a median age of 64 (49, 69) years. The most common symptom was cough, reported in 87.04% (262/301) of patients. At the time of admission, 60.13% (181/301) of patients presented with concurrent atelectasis. Among these cases, 72.93% (132/181) involved the right lung, and 50.28% (91/181) specifically affected the right middle lobe. Significant differences were observed between the atelectasis and non-atelectasis groups in several clinical variables, including age, proportion of patients working as farmers, incidence of night sweats, presence of lymphadenopathy with ring-like enhancement, involvement of the right main bronchus and right middle lobe bronchus, proportion of cases with involvement of two or more bronchial sites, and incidence of visible fistulae (*P* < 0.05). Demographic and clinical profiles are detailed in Table 1.

**TABLE 1:** Demographic and clinical characteristics between patients with and without atelectasis.

Variables	Atelectasis	No atelectasis	Standardize difference	*P*-value
*N*	181	120		
Age (years)	66.00 (55.00–70.00)	57.00 (32.75–67.00)	0.42 (0.18, 0.65)	<0.001*
Gender			0.17 (−0.06, 0.40)	0.142
Female	109 (60.22%)	62 (51.67%)		
Male	72 (39.78%)	58 (48.33%)		
Farmer			0.44 (0.21, 0.68)	<0.001*
No	77 (42.54%)	77 (64.17%)		
Yes	104 (57.46%)	43 (35.83%)		
Cough			0.02 (−0.21, 0.25)	0.874
No	23 (12.71%)	16 (13.33%)		
Yes	158 (87.29%)	104 (86.67%)		
Sputum			0.05 (−0.18, 0.28)	0.688
No	35 (19.34%)	21 (17.50%)		
Yes	146 (80.66%)	99 (82.50%)		
Fever			0.20 (−0.03, 0.43)	0.082
No	144 (79.56%)	85 (70.83%)		
Yes	37 (20.44%)	35 (29.17%)		
Hemoptysis			0.19 (−0.04, 0.42)	0.094
No	172 (95.03%)	108 (90.00%)		
Yes	9 (4.97%)	12 (10.00%)		
Chest pain			0.02 (−0.21, 0.25)	0.869
No	161 (88.95%)	106 (88.33%)		
Yes	20 (11.05%)	14 (11.67%)		
Dyspnea			0.14 (−0.09, 0.37)	0.239
No	118 (65.19%)	86 (71.67%)		
Yes	63 (34.81%)	34 (28.33%)		
Night sweats			0.27 (0.03, 0.50)	0.021*
No	162 (89.50%)	96 (80.00%)		
Yes	19 (10.50%)	24 (20.00%)		
Fatigue			0.17 (−0.06, 0.40)	0.144
No	171 (94.48%)	108 (90.00%)		
Yes	10 (5.52%)	12 (10.00%)		
Chest tightness			0.06 (−0.17, 0.29)	0.596
No	171 (94.48%)	115 (95.83%)		
Yes	10 (5.52%)	5 (4.17%)		
Tuberculous cavitation			0.04 (−0.19, 0.27)	0.734
No	165 (91.16%)	108 (90.00%)		
Yes	16 (8.84%)	12 (10.00%)		
Lymph node calcification			0.15 (−0.08, 0.38)	0.203
No	104 (57.46%)	60 (50.00%)		
Yes	77 (42.54%)	60 (50.00%)		
Lymphadenopathy with ring-like enhancement			1.13 (0.88, 1.37)	<0.001*
No	41 (22.65%)	86 (71.67%)		
Yes	140 (77.35%)	34 (28.33%)		
Right main bronchus involvement			0.31 (0.08, 0.54)	0.007*
No	160 (88.40%)	92 (76.67%)		
Yes	21 (11.60%)	28 (23.33%)		
Right intermediate bronchus involvement			0.18 (−0.06, 0.41)	0.132
No	154 (85.08%)	94 (78.33%)		
Yes	27 (14.92%)	26 (21.67%)		
Right upper lobe bronchus involvement			0.12 (−0.11, 0.35)	0.294
No	145 (80.11%)	90 (75.00%)		
Yes	36 (19.89%)	30 (25.00%)		
Right middle lobe bronchus involvement			0.48 (0.24, 0.71)	<0.001*
No	130 (71.82%)	108 (90.00%)		
Yes	51 (28.18%)	12 (10.00%)		
Right lower lobe bronchus involvement			0.01 (−0.22, 0.24)	0.947
No	164 (90.61%)	109 (90.83%)		
Yes	17 (9.39%)	11 (9.17%)		
Left main bronchus involvement			0.17 (−0.06, 0.40)	0.148
No	160 (88.40%)	99 (82.50%)		
Yes	21 (11.60%)	21 (17.50%)		
Left upper lobe bronchus involvement			0.16 (−0.07, 0.40)	0.170
No	139 (76.80%)	100 (83.33%)		
Yes	42 (23.20%)	20 (16.67%)		
Left lower lobe bronchus involvement			0.00 (−0.23, 0.23)	0.986
No	160 (88.40%)	106 (88.33%)		
Yes	21 (11.60%)	14 (11.67%)		
Involvement of at least two bronchial segments			0.24 (0.01, 0.48)	0.037*
No	143 (79.01%)	82 (68.33%)		
Yes	38 (20.99%)	38 (31.67%)		
Fistula visible			0.48 (0.25, 0.72)	<0.001*
No	141 (77.90%)	67 (55.83%)		
Yes	40 (22.10%)	53 (44.17%)		
Sputum smear			0.12 (−0.11, 0.35)	0.312
No	161 (88.95%)	102 (85.00%)		
Yes	20 (11.05%)	18 (15.00%)		
Sputum culture			0.13 (−0.10, 0.36)	0.265
No	140 (77.35%)	86 (71.67%)		
Yes	41 (22.65%)	34 (28.33%)		

*At the 0.05 level (double tailed), the correlation is significant.

### 3.2 Risk factors of atelectasis

To uncover atelectasis-related risks, a univariate logistic regression was performed on the entire sample. As shown in Table 2, eight variables were significantly associated with atelectasis: age, occupation as a farmer, presence of night sweats, lymphadenopathy with ring-like enhancement, involvement of the right main bronchus, involvement of the right middle lobe bronchus, involvement of two or more bronchial sites, and the presence of visible fistulae. These variables were subsequently included in a multivariable logistic regression analysis. The results demonstrated that increasing age, occupation as a farmer, lymphadenopathy with ring-like enhancement, and involvement of the right middle lobe bronchus were independent risk factors for atelectasis.

**TABLE 2:** Univariate and multivariable analysis of associations between candidate exposures and atelectasis.

Exposures	Univariate OR (95% CI)	*P*-value	Multivariable OR (95% CI)	*P*-value
Age (years)	1.02 (1.01–1.04)	<0.001*	1.03 (1.01–1.04)	0.003*
Farmer	2.42 (1.50–3.89)	<0.001*	1.85 (1.03–3.35)	0.041*
Night sweats	0.47 (0.24–0.90)	0.023*	0.64 (0.29–1.42)	0.275
Lymphadenopathy with ring-like enhancement	8.64 (5.09–14.64)	<0.001*	9.62 (5.21–17.77)	<0.001*
Right main bronchus involvement	0.43 (0.23–0.80)	0.008*	0.56 (0.25–1.26)	0.161
Right middle lobe bronchus involvement	3.53 (1.79–6.96)	<0.001*	5.32 (2.26–12.52)	<0.001*
Involvement of at least two bronchial segments	0.57 (0.34–0.97)	0.038*	0.88 (0.42–1.83)	0.728
Fistula visible	0.36 (0.22–0.59)	<0.001*	0.55 (0.28–1.07)	0.080

*At the 0.05 level (double tailed), the correlation is significant.

### 3.3 Development and verification of the prediction model

**FIGURE 2:**
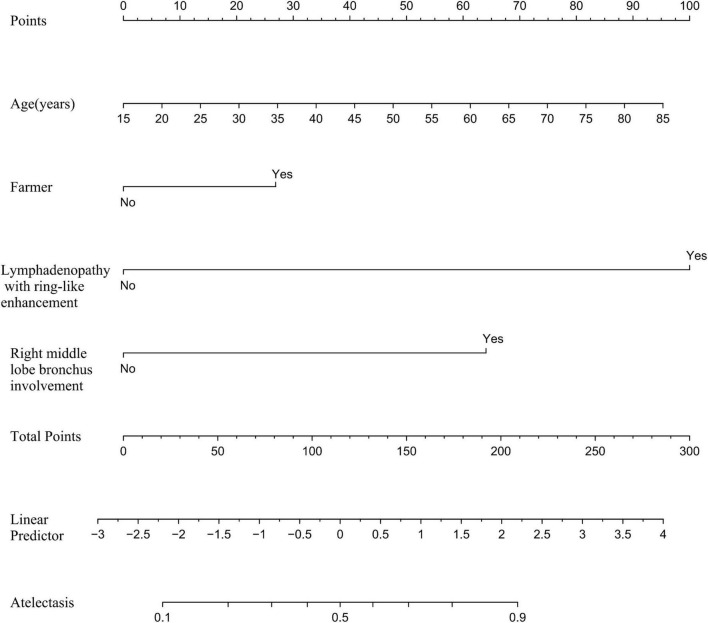
Nomogram to predict the probability of atelectasis in patients.

**FIGURE 3:**
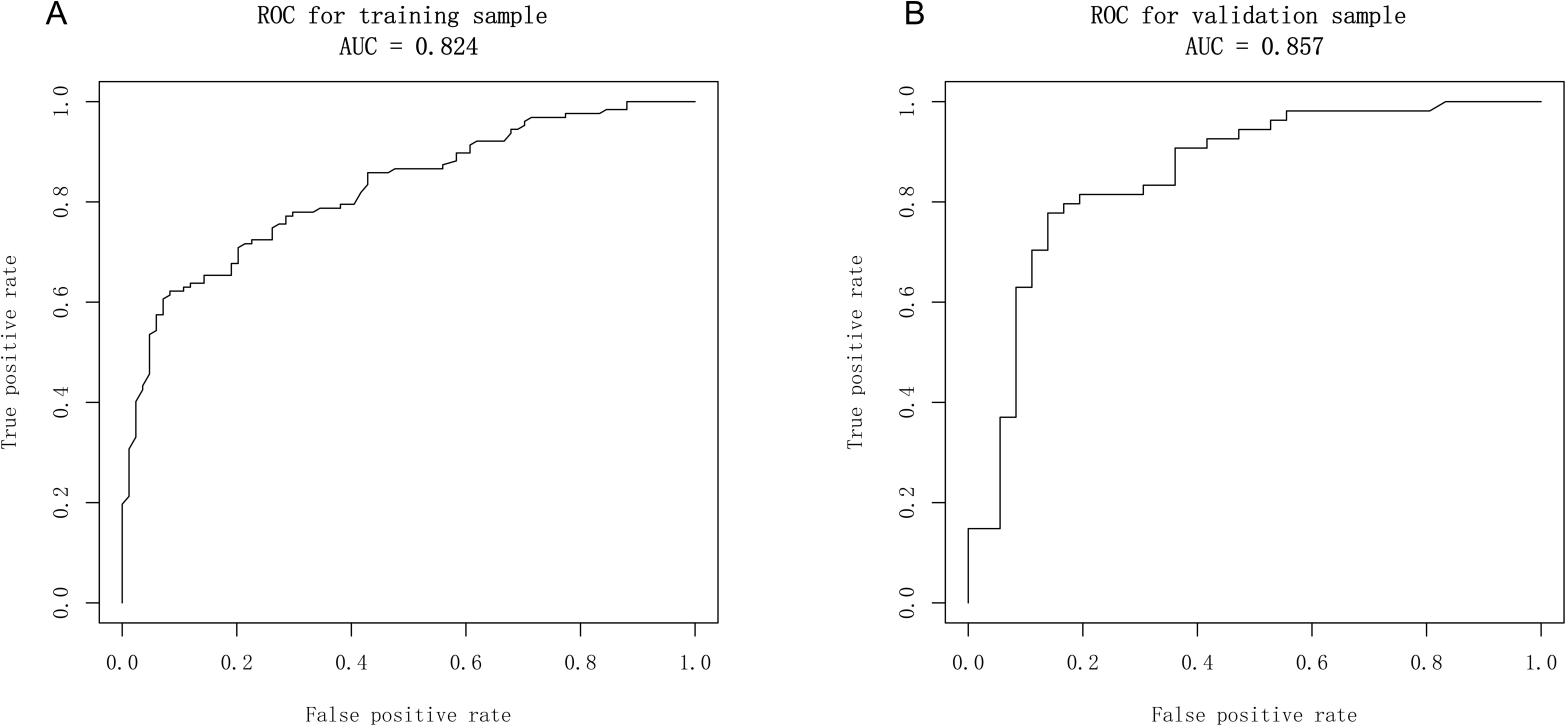
Receiver operator characteristic (ROC) curves of the nomogram. **(A)** Calibration plot of the nomogram in the training group. **(B)** Calibration plot of the nomogram in the validation group.

**FIGURE 4:**
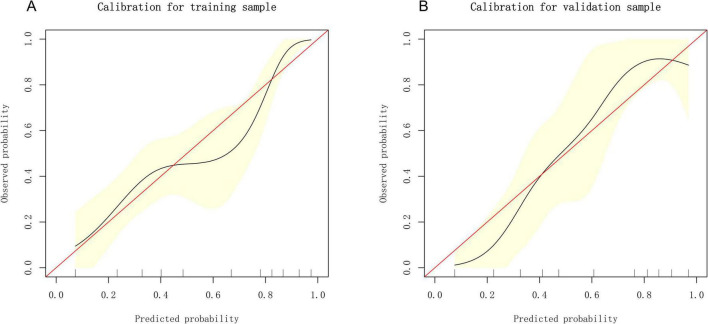
Calibration curves of the nomogram. **(A)** Calibration plot of the nomogram in the training group. **(B)** Calibration plot of the nomogram in the validation group.

**FIGURE 5:**
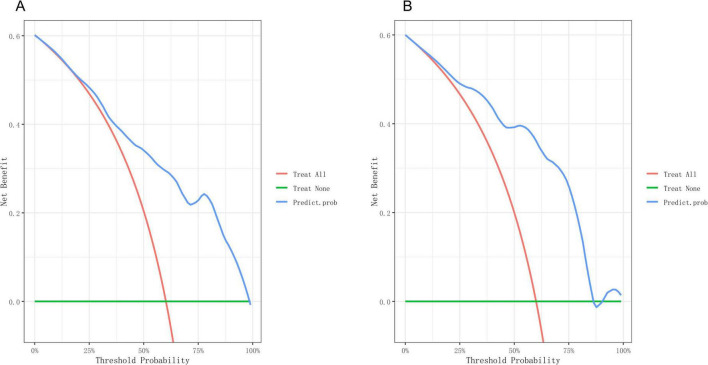
Decision curve of the nomogram. **(A)** Decision curves of the nomogram in the training group. **(B)** Decision curves of the nomogram in the validation group.

Based on the independent predictive variables identified by multivariable logistic regression analysis (age, occupation as a farmer, lymphadenopathy with ring-like enhancement, and involvement of the right middle lobe bronchus), a nomogram model was developed to predict the risk of atelectasis (Figure 2). Stepwise selection and correction for multicollinearity were used to ensure the statistical significance and clinical relevance of the selected variables. Lymph node enlargement with annular enhancement represented the dominant risk determinant. The model achieved favorable discrimination with training AUC of 0.824, improving to 0.857 upon validation (Table 3 and Figure 3), thus confirming predictive reliability. Calibration assessment via 500 bootstrap samples indicated optimal correspondence between predicted probabilities and observed events in both groups (Figure 4). Decision curve evaluation (Figure 5) substantiated the model’s clinical advantage compared to blanket treatment scenarios, with demonstrable net benefit observed at threshold probabilities of 0.2–0.9 in the derivation cohort and 0.15–0.85 in the validation cohort.

**TABLE 3:** Area under the curve of the nomogram prediction model.

Dataset cohort	AUC	95% CI of the AUC
Training cohort	0.824	0.685–0.866
Validation cohort	0.857	0.702–0.877
Entire cohort	0.833	0.784–0.877

## 4 Discussion

### 4.1 Background and significance of model development

The clinical importance of TBTB complicated by atelectasis is well recognized (2, 3). To overcome the limitations of relying solely on clinical judgment for risk stratification, this study developed a nomogram based on multivariable analysis to predict the risk of atelectasis in patients with lymph node fistula TBTB. The model incorporates easily obtainable variables, including demographic factors (age and occupation), clinical indicators (symptoms, sputum smear, and culture), and CT findings (bronchial lesions, enhancement patterns of mediastinal, and/or hilar lymph nodes), allowing for a multidimensional risk assessment without significantly increasing the burden of routine diagnosis and treatment. By organizing and quantifying scattered clinical data, the nomogram provides a visual tool to support personalized clinical decisions.

This model may help clinicians identify high-risk patients early, prioritize bronchoscopic evaluations, and adjust anti-tuberculosis regimens, while reducing unnecessary diagnostic procedures in low-risk patients. It may be particularly useful in primary care settings and areas with limited medical resources. Such multidimensional, evidence-based tools could support improved clinical management of TBTB, promote more efficient use of healthcare resources, and help reduce the public health burden of tuberculosis.

### 4.2 Key risk-factor analysis

Four independent predictors of atelectasis in TBTB were identified: increasing age, farming occupation, involvement of the right middle lobe bronchus, and mediastinal lymphadenopathy with ring-like enhancement. These factors span host characteristics, occupational exposures, local anatomical features, and radiological signs. The findings are consistent with previous studies (7, 12–14) and provide a more systematic understanding of the mechanisms underlying pulmonary atelectasis in lymph node fistula TBTB.

#### 4.2.1 Age

The study demonstrated that age alone significantly increased the likelihood of atelectasis in bronchial-tuberculosis patients. Multivariable analysis showed that the risk of atelectasis increased by approximately 3% with each additional year of age, aligning with previous findings reporting a 3.6% increase (OR = 1.036, 95% CI = 1.012–1.061, *P* = 0.003) (7). Aging leads to progressive depletion of glycosaminoglycans in bronchial cartilage and fragmentation of elastic fibers, which together reduce airway compliance (15–17). In addition, both ciliary beat frequency (18) and macrophage phagocytic activity decline with age (19), leading to the accumulation of tuberculous exudate and caseous debris within the airway lumen. This promotes “endogenous obstruction,” making older individuals more susceptible to irreversible airway collapse even under similar levels of tuberculous inflammation.

#### 4.2.2 Farming occupation

Occupational research indicates that sustained exposure to farm dust, sulfuryl-fluoride fumigants, and organophosphate pesticides increases airway-epithelium permeability and particle retention, resulting in airway injury (20, 21). Farming for 20 or more years has been associated with higher risks of chronic cough (OR = 1.52), wheezing (OR = 1.37), and dyspnea (OR = 1.83), with a clear dose–response relationship (12). This is consistent with the 1.85-fold increased risk observed in our cohort. However, chemical and dust exposure are only part of the explanation. Limited access to medical care and insufficient tuberculosis (TB) education in rural areas also delay the start of anti-TB treatment. One survey in China found that awareness of common TB symptoms was poor; 35% of respondents considered night sweats to be a normal sign of fatigue, leading to an average patient delay of 6.1 weeks from symptom onset to first medical contact (22). Another study reported that rural TB patients typically passed through 2.7 levels of the healthcare system before receiving standard treatment, with transfers from county hospitals to designated TB centers accounting for 58% of the delay (23). A similar pattern was seen in rural Brazil, where the average delay from symptom onset to specialist care was 9.4 weeks—3.2 weeks longer than in urban areas (24). These delays likely represent a critical period during which disease progression occurs, which may explain the stronger association between farming and atelectasis observed in this study.

#### 4.2.3 Involvement of the right-middle-lobe bronchus

The right middle lobe bronchus is long and narrow and joins the right main bronchus at an acute angle. These anatomical features impair mucus clearance and increase vulnerability to external compression (13, 25). It is also located near mediastinal lymph node stations 4R and 7, making it particularly prone to involvement in tuberculous lymphadenitis. Enlarged lymph nodes can directly compress the airway and reduce its structural integrity. In addition, caseous necrosis may rupture through the lymph node capsule and damage the bronchial wall (14). The resulting infiltration by CD4^+^ T cells and granulomatous inflammation further damages the mucosal and cartilaginous layers, reducing elasticity (26). The combined effects of compression and inflammation promote bronchial collapse, cause mucus retention in distal airways, and lead to secondary infection. This infection can, in turn, increase lymphatic drainage and worsen lymph node inflammation, creating a self-reinforcing cycle that predisposes the right middle lobe to atelectasis.

#### 4.2.4 Mediastinal lymphadenopathy with ring-like enhancement

Ring enhancement on contrast-enhanced CT, characterized by a hypoattenuating center surrounded by a hyperenhanced rim, is a hallmark of necrotic tuberculous lymphadenitis. The peripheral hyperenhancement reflects inflammatory hyperemia and neovascularization, while the non-enhancing center corresponds to caseous necrosis (14, 27). Once liquefaction occurs, necrotic material may breach the nodal capsule and erode the adjacent bronchial wall, leading to mural thickening, ulceration, or the formation of a bronchus-to-node fistula. Subsequent fibrotic healing can result in fixed airway stenosis (3). In this study, ring enhancement had the highest regression coefficient (β = 0.87), highlighting its key role in promoting bronchial collapse or narrowing. This sign was most commonly observed in lymph node stations 4R and 7. When present near the structurally vulnerable right middle lobe bronchus, the likelihood of complications increases. Early bronchoscopic evaluation is recommended in the presence of ring enhancement (28), and timely corticosteroid therapy or interventional procedures may interrupt the “necrosis → erosion → fibrosis” cascade. Thus, ring enhancement should be regarded as a critical imaging marker for predicting and preventing bronchial damage.

The nomogram developed in this study showed good discrimination in both the training and validation sets, with AUCs of 0.824 and 0.857, respectively. Sensitivity and specificity both exceeded 70%, and the overall accuracy approached 80%. This model may help clinicians accurately identify high-risk patients, reduce the occurrence of respiratory complications, and improve clinical outcomes. In summary, the four-factor model evaluates the risk of atelectasis in TBTB across four key domains: host susceptibility, occupational exposure, local anatomical vulnerabilities, and characteristic imaging features. The quantitative approach provides both theoretical and practical support for subsequent interventions and precision management.

### 4.3 Clinical implementation

Within 24 h of admission, the proposed model can stratify patient risk using standard enhanced CT scans and electronic medical records. The hospital information system can then automatically assign appropriate follow-up intervals. For surgical candidates identified as high risk, preoperative management—such as mediastinal lymph node debulking or bronchial stenting—may help reduce postoperative atelectasis. In primary care settings, occupational history and CT findings can be used to screen for high-risk individuals and expedite referral, enabling coordinated care across different levels of the healthcare system.

### 4.4 Limitations and future research directions

Several important limitations must be acknowledged, each of which also indicates a path for future work. First, the model was developed and internally validated using retrospective data from a single tertiary-care center, which may restrict its generalizability to other institutions, populations, and geographic regions. Although bootstrap validation showed good performance, the lack of external validation remains a critical shortcoming. Future studies should therefore perform prospective external validation in geographically diverse multicenter cohorts and, if necessary, recalibrate the model to ensure robustness and applicability.

Second, variability in CT scanner settings and inter-reader interpretation could compromise the model’s transportability. Subsequent studies should endeavor to employ standardized imaging protocols and explore AI-assisted analysis to minimize instrument- and observer-related variation.

Third, the predictor “occupation (farmer)” is likely a surrogate for unmeasured socioeconomic factors such as income, education, living conditions, nutritional status, healthcare access, and treatment delays, introducing residual confounding. Future research should systematically collect granular socioeconomic variables—including income, education, residential environment, and health-insurance status—to quantify and adjust for these potential confounders more accurately.

## 5 Conclusion

The nomogram established here showed excellent discrimination and prediction performance, enabling clinicians to pinpoint high-risk patients with greater precision. This may help reduce respiratory complications and improve patient outcomes. The four-factor model systematically addresses the mechanism of atelectasis in TBTB from multiple perspectives, including host vulnerability, occupational exposure, anatomical predisposition, and imaging features, thereby offering theoretical and empirical support for future targeted management strategies.

## Data Availability

The raw data supporting the conclusions of this article will be made available by the authors, without undue reservation.
